# The Long-Term Treatment of Drug-Resistant Migraine with the Modified Atkins Ketogenic Diet: A Single-Center, Retrospective Study

**DOI:** 10.3390/nu16244324

**Published:** 2024-12-15

**Authors:** Francesco Francini-Pesenti, Silvia Favaretto, Matteo D’Angelo, Martina Cacciapuoti, Lorenzo A Calò

**Affiliations:** 1 Clinical Nutrition Unit, DIDAS Medicina dei Sistemi, Azienda Ospedale-Università di Padova, 35128 Padua, Italy; matteo.dangelo@aopd.veneto.it; 2 Neurology Clinic, Department of Neuroscience, Azienda Ospedale-Università di Padova, 35128 Padua, Italy; silvia.favaretto@aopd.veneto.it; 3 Nephrology, Dialysis and Transplantation Unit, Department of Medicine, Azienda Ospedale-Università di Padova, 35128 Padua, Italy; martina.cacciapuoti@phd.unipd.it

**Keywords:** ketogenic diet, modified Atkins diet, headache, migraine prophylaxis

## Abstract

Despite advances in pharmacological therapies, migraine patients are often drug resistant. Further therapeutic options in this field are, therefore, desirable. Recent studies have highlighted the efficacy of ketogenic diet (KD) on improving migraine, but data on their long-term efficacy and safety are lacking. In this study, we retrospectively evaluated the long-term effectiveness of the modified Atkins ketogenic diet (MAD) in episodic or chronic drug-resistant migraine patients. 52 patients diagnosed with episodic or chronic drug-resistant migraine under modified Atkins ketogenic diet (MAD) were evaluated. In total, 41 patients followed the diet for 6 months and 33 for 12 months. After both 6 and 12 months, frequency, length, and intensity of migraine episodes, as well as the number of medications significantly decreased with respect to the start of the diet. Body mass index, high sensitivity PCR, diastolic blood pressure, fasting plasma insulin and HOMA index were also significantly reduced both after 6 and 12 months. No major metabolic changes were observed during MAD treatment. In conclusion, KD has been shown to be effective and safe in the long-term treatment of drug-resistant migraine. A high dropout rate still remains an important factor, which often limits its use.

## 1. Introduction

Migraine is a common neurological disease that affects about 12% of the general population [1], inducing disability, lost productivity, and increased medication use, causing enormous costs to societies [2]. It is characterized by recurrent episodes of moderate or severe pain, usually unilateral and of pulsating quality, and associated with nausea, vomiting, photophobia, and phonophobia.

According to the international Headache Society (IHS Classification ICHD-3), migraine can be divided into two main categories: migraine with aura and migraine without aura, the former defined by the presence of recurrent and reversible symptoms (typically visual, sensory, or language and speech impairment) associated with migraine attacks. Migraine is usually an episodic disorder, although in some cases, especially if untreated or inadequately treated, it can escalate to a particularly invalidating condition, which is chronic migraine [3].

The etiology of migraine is complex and multifactorial, involving genetic, behavioral and environmental factors [4]. A key role in the development of migraine attacks is played by the activation of the trigeminovascular nociceptive pathways, resulting in the release of neurochemical mediators such as calcitonin gene-related protein (CGRP) and pituitary adenylate cyclase activating polypeptide (PACAP), which can trigger migraine pain [5].

Recently, scientific research has been investigating the complex landscape of migraine comorbidities and their potential role on migraine pathophysiology and clinical expression [6]. Metabolic dysfunction and, in particular, insulin-resistance seem to be significantly associated with migraine, impacting attack severity and disability [6]. Indeed, while global prevalence of migraine is around 12% [1], in patients with metabolic syndrome it is estimated to be around 11.9% in males up to 22.5% in females [7]. It is hypothesized that altered systemic metabolic regulation, as in patients with insulin-resistance, may cause an imbalance in brain bioenergetic homeostasis, synaptic function, and neurotransmitter release [8]. The human brain has intense energetic requirements; its main energetic substrate is glucose, although other types of substrates, such as ketone bodies and lactate, may be used under certain circumstances [8]. Dysregulation in brain energy metabolism due to insulin resistance is believed to be associated with abnormal neuronal, astrocytic, and mitochondrial function, with altered transmembrane glucose transport and intracellular glycogen storage and reduced cerebral mitochondrial phosphorylation [8]. In addition, adipose tissue is considered a neuroendocrine organ, exhibiting an active metabolic activity with production of hormones, such as leptin, adiponectin, orexin, and adipocytokines, such as TNF-alpha and IL-6; these in turn can interfere with energy homeostasis pathways and create a pro-inflammatory milieu at both the systemic and CNS levels, potentially acting on central pain threshold, microglial cytokine production, and hypothalamic function [9].

The treatment of migraine is complex and constantly evolving. Nonsteroidal anti-inflammatory drugs (NSAIDs) and triptans are the most widely used drugs for acute attacks. NSAIDs cause numerous adverse effects, affecting gastrointestinal tract and kidney function, especially in prolonged use [10].

Patients with frequent and disabling attacks are advised to start prophylactic therapy, which mostly consists of specific medications that must be taken for months on end, with the aim of reducing the frequency and severity of migraine attacks. The first-line preventive drugs traditionally recommended by international guidelines can be divided into the following categories: tricyclic antidepressants, beta-blockers, calcium antagonists, antidepressants, and antiepileptics [11]. These drugs are often associated with a significant number of adverse events, impairing patient adherence to therapy, and are not recommended for everyone because of multisystem effects that can be harmful in patients with comorbidities. Second-line preventive therapy are onabotulinum toxin A, anti CGRP monoclonal antibodies, and more recently gepants, which are better tolerated but not suitable for all patients [12].

In this interesting and expanding scenario, a dietary approach for the prevention of migraine seems to be a promising option.

The ability of ketone bodies to be an efficient energy substrate of the central nervous system as an alternative to glucose [8] has led to speculation about the use of ketogenic diets (KDs) in the treatment of migraine. KDs are high-fat, moderate-protein, low-carbohydrate diets first used in the treatment of epilepsy. Because of the poor compliance of the classic KD, in which the ratio of lipids to carbohydrates plus protein is 4:1, several alternatives have been posed over time. These include the modified Atkins ketogenic diet (MAD), which requires weighing only carbohydrate-containing foods and allows a higher intake of protein foods [13]. The MAD, however, may raise concerns about the effects on the kidney of liberalized protein intake. In a recent systematic review and meta-analysis that summarized the main clinical studies on the topic, no study extended treatment with KD beyond 3 months [14]. Moreover, although these studies reported the efficacy of KD in improving migraine manifestations, they generally involved a small number of patients, were mostly limited to episodic migraine, and had a short observation time (usually up to three months). To date, data on the efficacy, tolerability, and safety of the long-term KDs in both episodic and chronic migraine patients are lacking. Therefore, in this study we aimed to evaluate the long-term effectiveness of the MAD in the treatment of drug-resistant episodic and chronic migraine.

## 2. Materials and Methods

### 2.1. Study Population

A single-center, retrospective, non-randomized study was conducted to evaluate the KD in the prevention of resistant migraine. According to the European Headache Federation, subjects were considered “resistant” if they experienced at least 3 months of eight or more debilitating headache days monthly, despite attempting at least three classes of preventive medication, which have proven ineffective, intolerable, or contraindicated [15].

Fifty-two patients previously treated at the Headache Center of Neurology Clinic of Azienda Ospedale-Università di Padova and diagnosed with episodic or chronic drug-resistant migraine were observed in the outpatient Nutrition Clinic of Azienda Ospedale-Università of Padua from January 2021 to May 2023, in order to start dietary treatment. Twelve patients were also followed at the outpatient clinic of the Nephrology, Dialysis and Transplantation Unit of Azienda Ospedale-Università of Padua due to their estimated Glomerular Filtration Rate (eGFR) between 60 and 89 mL/min/1.73 m^2^, likely due to the prolonged use of NSAIDs.

Patients with headache occurring 15 or more days/month for more than 3 months were considered as affected by chronic migraine. If headache occurred for less than 15 days/month, episodic migraine was diagnosed.

Exclusion criteria were pregnancy, other neurological diseases, cancer, liver failure, chronic kidney disease stage ≥ 3, insulin-treated diabetes mellitus, and psychosis.

### 2.2. Ethical Approval

The study was conducted in accordance with the principles expressed in the Declaration of Helsinki. The study protocol was approved by the Territorial Ethics Committee Central Area-EAST Veneto on 22 February 2024 (CET-ACEV:458n/AO/24). All patients provided written informed consent.

### 2.3. Protocol and Dietary Intervention

During the first visit, patients were offered the treatment with MAD. MAD is a high-fat and moderate-protein diet that provides up to 30 g carbohydrate per day and requires weighing only carbohydrate-containing foods [13]. This diet does not restrict foods containing only protein and fat (e.g., meat, cheese, eggs, oil). These characteristics promote treatment adherence better than other KDs. In the MAD the fat to protein plus carbohydrate ratio is about 1.2:1.

### 2.4. Migraine and Metabolic Evaluation

For all participants, migraine assessment and therapies, body weight, systolic blood pressure (SBP), diastolic blood pressure (DBP), and laboratory data were collected before the start of dietary treatment and then after 1, 3, 6, 9, and 12 months. In this paper, we have reported data before the start of MAD (T0), at 6 months (T1), and at 12 months (T2) of treatment.

Each patient was asked to record in a headache diary migraine attack frequency (days/month), duration (hours), and intensity assessed by a numeric rating scale (0 = no pain, 10 = worst possible pain) and quantity of analgesic tablet intake (number per month). Migraine-related disability was assessed with the Migraine Disability Assessment (MIDAS) questionnaire. Laboratory tests, measured after an overnight fast, included the following: fasting plasma glucose, fasting plasma insulin, serum triglycerides (TG), total cholesterol (TC), high-density lipoprotein cholesterol (HDL-C), low-density lipoprotein cholesterol (LDL-C), alanine aminotransferase (ALT), aspartate aminotransferase (AST), plasma uric acid, and high-sensitivity C-reactive protein (hs-CRP). To assess IR, homeostatic model assessment (HOMA-IR) was calculated using the formula: HOMA-IR = [fasting glucose (mmol/L) × insulin (μU/mL)/22.5] [14]. IR was defined as HOMA-IR ≥ 2.5, according to NHLBI/AHA criteria [16]. All patients were asked to monitor ketonuria daily using a commercial semi-quantitative colorimetric urinary stick and to record the result (low = +, moderate = ++, and high = +++).

### 2.5. Statistical Analysis

Data were reported as mean ± standard deviation (SD) for continuous variables and frequency for categorical variables. To determine whether continuous data were normally distributed we used the Shapiro–Wilk test. A paired *t*-test or the Wilcoxon test was used to compare differences between continuous variables in the MAD group. An unpaired *t*-test was used to compare differences between subgroups of patients.

## 3. Results

**Figure 1:**
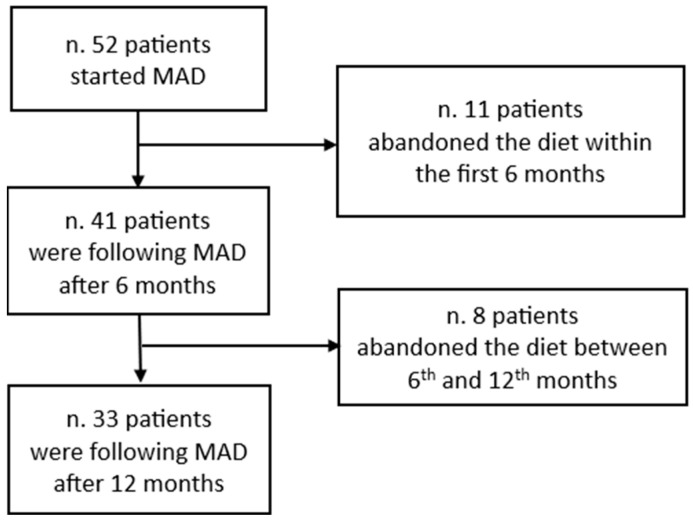
Patient flow diagram.

The patient flow diagram of patients observed through the study is shown in Figure 1.

Table 1 shows the general characteristics of the 52 patients who started MAD.

In total, 45 (87%) patients were females and 7 (13%) were males. A total of 28 patients (54%) suffered from chronic migraine and 24 (46%) from episodic migraine. Twelve (23%) patients reported episodes of aura.

At the start of treatment (T0), the mean body mass index (BMI) was 27.9 ± 6.8 kg/m^2^, 16 patients were obese (BMI ≥ 30 kg/m^2^), and 9 were overweight (BMI 25–29.9 kg/m^2^). The mean frequency of migraine attacks was 14.3 ± 8.1 days per months, the mean length of each attack was 17.0 ± 14.5 h per day, and intensity of pain was 8.3 ± 1.4. The mean MIDAS score was 94.4 ± 53.1 points. Before the start of the MAD, medication intake for migraine attacks was 20.7± 15.1 doses per month. All patients reported using drugs for the acute migraine attack. A total of 13 subjects (25%) were taking NSAIDs 15 or more days per month, and 8 (15%) were taking triptans or their combinations 10 or more days per month. Therefore, at the first visit, medication overuse headache (MOH) occurred in about 40% of the entire sample. 

At six months of the MAD (T1), 41 patients were still following the MAD. In this group, the number of episodes per week, their duration and intensity, the number of medications per month, and the MIDAS score were significantly reduced (Table 2).

Of this group, seventeen patients (41%) were classifiable with MOH at T0 and none at T1. BMI, hsCRP, DBP, fasting plasma insulin, and HOMA index were also significantly reduced. The lipid profile showed a reduction in triglycerides and an increase in HDL-cholesterol plasma values. The mean uric acid level increased non-significantly, remaining within normal limits. Total cholesterol and LDL-cholesterol did not change significantly. 

To assess the possible influence of weight changes on migraine, we divided patients who followed MAD up to 6 months into two subgroups, according to BMI loss (subgroup A: <1 kg/m^2^, *n* = 15; subgroup B: ≥1 kg/m^2^, *n* = 26). The averages of frequency, duration, and intensity of attacks of headache, MIDAS, and hsCRP did not differ significantly between two groups at 6 months (Table 3).

After 12 months of the MAD (T2), 33 patients were still on diet. Compared to T1, BMI and DBP were significantly reduced, while the other parameters showed no significant changes (Table 4).

Thirteen patients (39%) reported MOH at T0 and none at T2.

Nine patients with CKD-G2 were treated with MAD for 6 months and seven patients for 12 months. In these patients, eGFR did not change (from 72.6 ± mL/min to 71.9 ± mL/min (n.s.) in the former nine patients and from 73.8 ± 5.9 mL/min to 73.3 ± 5.2 (n.s.) in the latter seven patients.

All patients reported positive ketonuria on most treatment days, both after 6 and 12 months. Eleven patients abandoned the diet within the first 6 months and 8 during the next 6 months. Of the 11 patients who abandoned the diet within the first 6 months, 6 complained of excessive appetite and carbohydrate cravings, 4 due to keto-flu and 1 due to keto-rash. All eight patients who abandoned the diet after 6 months reported reduced food choices, incompatible with a normal social life, as the reason for discontinuing the diet.

## 4. Discussion

In this study, we described the long-term effects of the MAD in preventing migraine attacks.

After 6 months, patients following MAD showed a significant and considerable improvement in migraine manifestations, resulting in reduced disability as assessed by the MIDAS questionnaire.

Patients who were still following the MAD after 12 months showed essentially unchanged clinical ameliorations compared with the first 6 months, demonstrating that the effects of the KD were maintained over time.

Previous studies have reported the effectiveness of KDs in the treatment of migraine, but their observation period was limited to a few weeks or months. Most of these studies used the very low-calorie ketogenic diet (VLCKD) and MAD [17,18,19,20,21,22,23]. When KD was compared with non-ketogenic diets, the former proved to be more effective in reducing attack frequency and tablet use [22].

KDs are known to cause weight loss by inducing a reduction in appetite. In turn, weight loss can improve migraine itself [24]. Consequently, the improvement in symptoms observed during MAD could be attributed to weight loss due to its anorectic effect, although the diet administered in our study was not hypocaloric per se. Therefore, we divided the patients according to whether they lost less or more than 1 kg/m^2^ BMI. No significant difference in clinical outcomes was observed between the two groups, suggesting that the effects of the KD were not primarily attributable to weight loss.

As a consequence of symptom improvement, the consumption of drugs for the migraine attack medications, such as NSAIDs and triptans, was lowered. Before treatment with KG, more than 40% of patients fell within the criteria of MOH. Chronic use of NSAIDs increases the risk of gastrointestinal, renal, and cardiovascular complications, mainly due to the inhibition of the enzyme cyclooxygenase-1, interfering with the production of prostaglandins that play a role in protecting the gastric mucosa and in renal hemodynamics [24]. Long-term exposure to high doses of NSAIDs is associated with a significant risk of acute and chronic kidney disease, even in young and middle-aged adults [25].

It has been hypothesized that inflammation and insulin resistance are among the mechanisms involved in the pathogenesis of migraine [6,26]. Beta-hydroxybutyrate (BHB), the main ketone body in plasma during KD, inhibits inflammatory signaling involved in several chronic diseases. One of the main targets of the BHB is the NLRP3 inflammasome [27], a multiprotein complex activated in the central nervous system at the onset of migraine attacks [28].

In our study, MAD induced a small but significant reduction in hsCRP after 6 months of dietary treatment, maintained even after 12 months in those who had continued the diet, confirming the anti-inflammatory effect as a conceivable mechanism to explain the effects of ketosis on migraine. After 6 and 12 months of the MAD, the HOMA index was reduced compared to t0. It is noteworthy that IR and inflammatory response are interlinked, and inflammation plays a key role in the development of IR. In particular, overnutrition, through increased blood glucose and lipids, induces the production of pro-inflammatory mediators that, in turn, interfere with insulin signaling pathways [29].

KD can improve IR through several ways. BHB stimulates AMP-activated protein kinase (AMPK), an energy sensor that regulates cellular metabolism (20). AMPK activation reduces inflammasome formation and oxidative stress [30] and promotes glucose uptake, insulin sensitivity, fatty acid oxidation, and mitochondriogenesis [31]. However, data available in the literature have only been shown that low-carb diets and KDs improve fasting insulin sensitivity, while some studies showed a neutral effect [32,33] or a worsening of glucose tolerance [34].

In this study, we observed a high dropout rate during KD treatment. In total, 23% of patients who started treatment dropped out after 6 months and 37% after 12 months. These data were similar to those reported in a recent review on this topic [14]. In our patients, poor compliance with the diet was due to reduced food choice, incompatible with a normal social life, and carbohydrate craving, especially in the first weeks of the diet. More rarely, patients stopped the MAD because of keto-flu, a set of symptoms including brain fog, dizziness, irritability, food craving, and nausea, which can occur in the first few days after starting the KD and can last from a week to a month [35]. Food price may have been another factor favoring the high dropout rate [36,37]. In fact, high-protein foods (meat, fish, cheese, etc.) are more expensive than grains (bread, pasta, rice, etc.).

Some adverse effects attributable to MAD were observed in our sample. The most frequent was constipation, due to the reduction in fruits and vegetables and was treated with dietary fiber supplements. Two patients experienced keto-rash, also called prurigo pigmentosa, a rare skin manifestation characterized by a red, itchy rash around the trunk and neck [38]. In one case, the symptoms regressed after complete discontinuation of the diet and in the other after a modest increase in carbohydrate intake (from 30 to 60 g per day) that did not completely inhibit ketosis.

Lipid alterations during KDs are inconsistently reported in the literature [39]. In our study, we observed minimal but significant changes at 6 and 12 months for HDL-cholesterol, which increased, and for triglycerides, which decreased.

Hyperuricemia has been reported to be a metabolic alteration induced by the KD [40]. During ketosis, proximal tubular reabsorption of uric acid is inhibited by acetoacetic and beta-hydroxybutyric acid [41], which may result in the increase in its plasma levels. Our data showed a slight but nonsignificant increase in uric acid after 6 and 12 months of the MAD. No patient had to discontinue diet or take medications because of an increase in plasma uric acid level. After 6 months of the MAD we observed a reduction in BP, significant only for DP. To date, there is no conclusive data about the effects of KD on BP in the scientific literature [42]. Several mechanisms may explain the reduction in BP during ketosis. Body weight loss, per se, can indirectly induce BP reduction. In addition, KD reduces plasma insulin, promoting sodium and water loss [43] and affecting the hypothalamic–pituitary–adrenal axis and the sympathetic nervous system [44].

In the patients with CKD, the MAD may raise some concerns due to increased protein intake. Twelve of our patients had stage 2 CKD, and they started the KD with the intention of reducing the use of NSAIDs. A treatment with the MAD, a high protein KD, could have been considered inappropriate in these patients. However, in stage 2 kidney disease, protein restriction is not recommended [45] and the reduction in NSAIDs could bring more benefits than reducing protein intake. Furthermore, ketone bodies have many physiological effects and are even suggested to exert renoprotective effects [46]. Consistent with these considerations, plasma creatinine and eGFR levels remained substantially unchanged in all these patients at both 6 and 12 months.

Our study has some limitations, including its uncontrolled retrospective design and small sample size. On the other hand, it is the first study to evaluate long-term KD in drug-resistant migraine patients by extending the observation to one year. In addition, all patients treated were from the same headache center.

## 5. Conclusions

In conclusion, MAD represents an alternative approach to treat migraine, especially in patients resistant to drug therapy. In our study, this treatment proved safe even in the long term, although burdened by a high dropout rate, an aspect that seems to be its main limitation. The positive metabolic changes observed in lipid profile, insulin resistance, inflammation, and blood pressure may represent additional benefits of this diet. Further controlled trials are required to confirm our results.

## Figures and Tables

**Table 1:** General characteristics of the patients who started MAD.

Number of patients	52
Age, years	44.3 ± 10.5
Males, *n* (%)	7 (13)
BMI, kg/m^2^	27.9 ± 6.8
Episodic migraine, *n*	24
Chronic migraine, *n*	28
Migraine with aura, *n* (%)	12 (23)

**Table 2:** Effects of MAD after 6 months of treatment on migraine, BMI, blood pressure, and laboratory parameters. Abbreviations: BMI, Body Mass Index; hsCRP, high-sensitivity C-Reactive Protein; DBP, Diastolic Blood Pressure; HDL, High-Density Lipoproteins; HOMA, Homeostatic Model Assessment; MIDAS, Migraine Disability Assessment Score Questionnaire; LDL, Low-Density Lipoproteins; SBP, Systolic Blood Pressure.

	T0	T1
Attack frequency (number/month)	13.8 ± 9.0	4.7 ± 3.2 (*p* < 0.001)
Pain intensity	8.2 ± 1.4	4.3 ± 1.0 (*p* < 0.001)
Length of each attack (hours)	9.5 ± 5.1	7.2 ± 5.7 (*p* < 0.005)
Analgesic drug intake (n/week)	18 ± 16	5 ± 4 (*p* < 0.001)
MIDAS score	97 ± 56	24 ± 11 (*p* < 0.001)
BMI (kg/m^2^)	28.5 ± 6.8	27.7 ± 5.9 (*p* < 0.001)
SBP (mmHg)	123 ± 14	119 ± 20 (n.s. *)
DBP (mmHg)	83 ± 9	81 ± 8 (*p* < 0.001)
HOMA index	3.8 ± 2.6	2.8 ± 1.6 (*p* < 0.05)
Uric acid (μmol/L)	5.0 ± 0.9	5.1 ± 1.0 (n.s. *)
Total cholesterol (mg/dL)	186 ± 28	187 ± 25 (n.s. *)
HDL-cholesterol (mg/dL)	59± 10	60 ± 9 (*p* < 0.05)
LDL-cholesterol (mg/dL)	109 ± 26	110 ± 25 (n.s. *)
Triglycerides (mg/dL)	88 ± 31	84 ± 24 (*p* < 0.05)
hsCRP (mg/L)	2.4 ± 1.0	2.0 ± 0.8 (*p* < 0.001)

* n.s.: non-significant.

**Table 3:** Changes in clinical parameters and hsCRP in patients who lost <1 kg/m^2^ (subgroup A) or ≥1 kg/m^2^ (subgroup B) after 6 months of treatment with MAD compared with T0. Abbreviations: hsCRP, high-sensitivity C-Reactive Protein; DBP, Diastolic Blood Pressure; HDL, High-Density Lipoproteins; HOMA, Homeostatic Model Assessment; MIDAS, Migraine Disability Assessment Score Questionnaire; LDL, Low-Density Lipoproteins; SBP, Systolic Blood Pressure.

	Difference T0–T1	
	Subgroup A * (*n* = 15)	Subgroup B ** (*n* = 26)
Attack frequency (number/month)	−10.1 ± 7.4	−8.6 ± 7.8 (n.s. ^§^)
Pain intensity	−3.9 ± 0.9	−4.0 ± 0.9 (n.s. ^§^)
Length of each attack (hours)	−0.9 ± 5.9	−3.5 ± 6.9 (n.s. ^§^)
Analgesic drug intake (n/week)	−12 ± 5	−14 ± 6 (n.s. ^§^)
MIDAS score	−68 ± 57	−74 ± 41 (n.s. ^§^)
BMI (kg/m^2^)	0.2 ± 0.5	−1.9 ± 1.2 (n.s. ^§^)
SBP (mmHg)	−3	−4 (n.s. ^§^)
DBP (mmHg)	−2	−2 (n.s. ^§^)
HOMA index	−0.8 ± 1.0	−1.4 ± 1.2 (n.s. ^§^)
Uric acid (μmol/L)	0.2 ± 0.5	0.1 ± 0.4 (n.s. ^§^)
Total cholesterol (mg/dL)	3 ± 5	−1 ± 6 (n.s. ^§^)
HDL-cholesterol (mg/dL)	1 ± 3	3 ± 5 (n.s. ^§^)
LDL-cholesterol (mg/dL)	2 ± 4	−1 ± 6 (n.s. ^§^)
Triglycerides (mg/dL)	−3 ± 6	−4 ± 7 (n.s. ^§^)
hsCRP	−0.2 ± 0.3	−0.7 ± 0.5 (n.s. ^§^)

* Subgroup A: patients who lost <1 kg/m^2^. ** Subgroup B: patients who lost ≥1 kg/m^2^. ^§^ n.s.: non-significant.

**Table 4:** Characteristics of migraine attacks BMI, blood pressure, and laboratory parameters after 6 and 12 months of MAD (*n* = 33 patients). Abbreviations: BMI, Body Mass Index; hsCRP, high-sensitivity C-Reactive Protein; DBP, Diastolic Blood Pressure; HDL, High-Density Lipoproteins; HOMA, Homeostatic Model Assessment; MIDAS, Migraine Disability Assessment Score Questionnaire; LDL, Low-Density Lipoproteins; SBP, Systolic Blood Pressure.

	T0	T1	T2
Attack frequency (number/month)	14.6 ± 9.5	3.9 ± 2.5 (*p* < 0.001)	3.7 ± 2.3 (n.s.) *
Pain intensity	8.1 ± 1.5	4.2 ± 1.0 (*p* < 0.001)	4.1 ± 0.8 (n.s.) *
Length of each attack (hours)	9.6 ± 5.4	6.9 ± 4.5 (*p* < 0.05)	6.8 ± 3.9 (n.s.) *
Analgesic drug intake (n/week)	18 ± 17	4.7 ± 4 (*p* < 0.001)	5 ± 4 (n.s.) *
MIDAS score	96 ± 59	24 ± 11 (*p* < 0.001)	22.8 ± 9.7 (n.s.) *
BMI (kg/m^2^)	29.3 ± 6.9	28.5 ± 5.9 (*p* < 0.05)	28.8 ± 5.8 (n.s.) *
SBP (mmHg)	123 ± 15	121 ± 12 (*p* < 0.01)	121 ± 10 (n.s.) *
DBP (mmHg)	83 ± 10	81 ± 8 (*p* < 0.001)	80 ± 7 (*p* < 0.05) *
HOMA index	3.9 ± 2.7	2.8 ± 1.6 (*p* < 0.05)	2.6 ± 1.4 (n.s.) *
Uric acid (μmol/L)	5.0 ± 0.9	5.1 ± 1.0 (n.s.)	5.2 ± 0.9 (n.s.) *
Total cholesterol (mmol/L)	185 ± 30	186± 27 (n.s.)	187.± 23 (n.s.) *
HDL-cholesterol (mg/dL)	58 ± 11	59 ± 10 (*p* < 0.05)	59 ± 9 (n.s.) *
LDL-cholesterol (mg/dL)	110 ± 27	110 ± 25 (n.s.)	112 ± 21 (n.s.) *
Triglycerides (mg/dL)	86 ± 30	82 ±23 (*p* < 0.05)	83 ± 21 (n.s.) *
hsCRP (mg/L)	2.5 ± 1.0	2.1 ± 0.8	2.1 ± 0.6

* n.s.: non-significant; significance level with respect to T1.

## Data Availability

The data presented in this study are available on request from the corresponding author due to the need to save patients’ privacy.
